# Electron-Beam Irradiation Induced Regulation of Surface Defects in Lead Halide Perovskite Thin Films

**DOI:** 10.34133/2021/9797058

**Published:** 2021-06-02

**Authors:** Binbin Jin, Ding Zhao, Fei Liang, Lufang Liu, Dongli Liu, Pan Wang, Min Qiu

**Affiliations:** ^1^Key Laboratory of 3D Micro/Nano Fabrication and Characterization of Zhejiang Province, School of Engineering, Westlake University, 18 Shilongshan Road, Hangzhou, 310024 Zhejiang Province, China; ^2^Institute of Advanced Technology, Westlake Institute for Advanced Study, 18 Shilongshan Road, Hangzhou, 310024 Zhejiang Province, China; ^3^State Key Laboratory of Crystal Materials and Institute of Crystal Materials, Shandong University, Jinan 250100, China; ^4^State Key Laboratory of Modern Optical Instrumentation, College of Optical Science and Engineering, Zhejiang University, Hangzhou 310027, China

## Abstract

Organic-inorganic hybrid perovskites (OIHPs) have been intensively studied due to their fascinating optoelectronic performance. Electron microscopy and related characterization techniques are powerful to figure out their structure-property relationships at the nanoscale. However, electron beam irradiation usually causes damage to these beam-sensitive materials and thus deteriorates the associated devices. Taking a widely used CH_3_NH_3_PbI_3_ film as an example, here, we carry out a comprehensive study on how electron beam irradiation affects its properties. Interestingly, our results reveal that photoluminescence (PL) intensity of the film can be significantly improved along with blue-shift of emission peak at a specific electron beam dose interval. This improvement stems from the reduction of trap density at the CH_3_NH_3_PbI_3_ surface. The knock-on effect helps expose a fresh surface assisted by the surface defect-induced lowering of displacement threshold energy. Meanwhile, the radiolysis process consistently degrades the crystal structure and weaken the PL emission with the increase of electron beam dose. Consequently, the final PL emission comes from a balance between knock-on and radiolysis effects. Taking advantage of the defect regulation, we successfully demonstrate a patterned CH_3_NH_3_PbI_3_ film with controllable PL emission and a photodetector with enhanced photocurrent. This work will trigger the application of electron beam irradiation as a powerful tool for perovskite materials processing in micro-LEDs and other optoelectronic applications.

## 1. Introduction

Organic-inorganic hybrid perovskites (OIHPs) have emerged as a group of promising optoelectronic materials due to their large light absorption coefficient [1–3], high carrier mobility, and long carrier diffusion length [4–8]. The power conversion efficiency (PCE) of OIHP solar cells has been improved from 3.8% to 25.5%, achieving comparable efficiency to the champion PCE of crystalline silicon-based counterparts [9, 10]. However, OIHP such as MAPbI_3_ (MA = CH_3_NH_3_^+^) are vulnerable to a variety of environmental factors including moisture [11–13], oxygen [14–16], heat [17, 18], and light irradiation [19, 20], which usually deform the crystal lattice or result in chemical decomposition. Defect passivation at the surfaces and grain boundaries (GBs) plays an important role in minimizing nonradiative recombination and maintaining stability of solution-processed polycrystalline OIHP films [21–24].

Diverse methods have been proposed to improve optoelectronic properties of OIHPs [25–31]. For instance, posttreatment in a certain humidity could enhance the PL of OIHP films, which is attributed to the partial solvation of methylammonium component and the formation of hydrogen bonding between the hydroxyl in water and uncoordinated halide ions [32]. More recently, light soaking using a standard 1-sun (100 mW/cm^2^) source and continuous laser even ultrafast pulsed laser (nanosecond and femtosecond laser) was reported to ameliorate perovskite film quality through the uniform lattice expansion [33], the release of residual stress [34], and surface polishing [35], respectively. The coexistence of light socking and oxygen can create a favorable environment to improve optical performance (enhancement in PL intensity) of perovskites through suppression of deep trap states by transferring photo-induced electrons from perovskites to the adsorbed oxygen [36–38]. A focused electron beam (e-beam) provided in a scanning electron microscope (SEM) or transmission electron microscope (TEM) can also be used to regulate crystal structures, as well as optoelectronic properties of materials [39–41]. Although a single-crystal MAPbBr_3_ microplate photodetector with increased photocurrent has been fabricated through direct e-beam exposure, it is widely accepted that e-beam irradiation can significantly degrade perovskite structures as well as the PL emission [42, 43]. Therefore, how electrons affect or interact with OIHPs is still an open question.

In the present work, we comprehensively study the effect of e-beam irradiation on MAPbI_3_ film properties. We find that the PL intensity firstly increases and then decreases with e-beam dose. Its maximum is around sixfold larger than that of unexposed region. We determine that improved PL is due to the knock-on effect of bombarding electrons at the MAPbI_3_ surface, which causes exposure of a fresh surface with less trap density, while the radiolysis plays a major role in deteriorating crystal structures and thus weakens PL emission. It is believed that e-beam exposure at some point is helpful to attain perovskite films with higher quality, and that device performance improves accordingly.

## 2. Results and Discussion

We choose MAPbI_3_ as a suitable candidate to study the e-beam irradiation induced structural and compositional changes since it is a promising photovoltaic material and more sensitive to e-beam than all inorganic perovskites [43]. MAPbI_3_ films were prepared on ITO glass using a one-step spin-coating process (see Method). X-ray diffraction (XRD) spectrum (Figure S1a) shows dominant diffraction peaks at 14.1°and 28.5°, which can be well indexed to (110) and (220) planes of tetragonal crystal structures. The absorption and PL spectra are shown in Figure S1b and S1c. The band edge and PL emission peak at around 762 nm were consistent with the previous research. Time-resolved PL (TRPL) decay profile of MAPbI_3_ was monitored to gain insight into charge carrier dynamic (Figure S1d), which displayed two delay life-time of *τ*_1_ = 14.7 ns and *τ*_2_ = 134.1 ns. All of these characterizations demonstrate the high quality of the synthesized MAPbI_3_ films.

**Figure 1:**
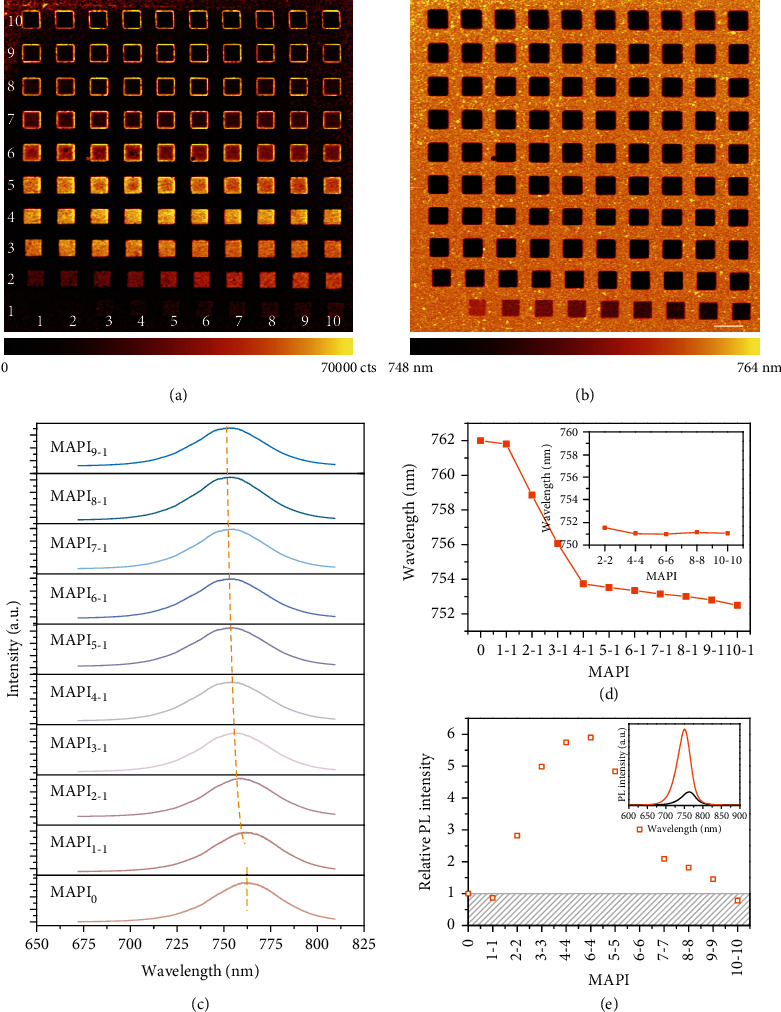
PL intensity of MAPI films irradiated by e-beam with various dose. (a) PL intensity mapping of MAPI arrays irradiated by e-beam, named as MAPI_1-1_ to MAPI_10-10_. Dose for MAPI_1-1_ is 50 *μ*C/cm^2^ and increased by 250 *μ*C/cm^2^ for the next square. (b) PL emission peak mapping of MAPI_1-1_ to MAPI_10-10_. Scale bar: 5 *μ*m. (c) PL emission spectra of MAPI_0_ to MAPI_9-1_. (d) Relationship between PL emission peak and e-beam dose. (e) Relationship between PL emission intensity and e-beam dose. Inset shows PL spectra of MAPI_0_ (black) and MAPI_6-4_ (orange).

A 10 by 10 square array with a side length of 4 *μ*m and a period of 10 *μ*m was exposed by a focused e-beam on the MAPbI_3_ film. Each square was named as MAPI_*m*-*n*_, where *m* defined the number of columns and *n* defined the number of rows. The acceleration voltage and basic area dose were 10 kV and 100 *μ*C/cm^2^. The dose factor for the first square (named as MAPI_1-1_) was 0.5 and increased by 2.5 for the next square (dose = area dose × dose factor = 100 *μ*C/cm^2^ × [(*m* − 1) × 25 + (*n* − 1) × 2.5 + 0.5]). That is to say, MAPI_1-1_ was irradiated by a dose of 50 *μ*C/cm^2^, and the dose for MAPI_10-10_ was increased to 24800 *μ*C/cm^2^ (see details of dose distribution in Figure S2). We first examined the PL intensity of e-beam-irradiated MAPI samples. 532 nm laser with a low power density of 0.2 mW/cm^2^ was employed to reduce photodamage. To our surprise, the PL intensity increases rapidly with the increase of irradiation dose (Figure 1(a)). MAPI_6-4_ displayed the largest PL emission (Figure 1(e)), which was around sixfold increase relative to that of unexposed sample (MAPI_0_). However, as the dose continues increasing, the PL intensity begins to attenuate and it is finally lower than that of MAPI_0_. The detailed PL emission spectra of MAPI_1-1_, MAPI_2-2_ to MAPI_10-10_ (along the diagonal of array) are shown in Figure S3.

Interestingly, PL intensity at the edge of the square before MAPI_6-4_is weaker than that at the center, while after MAPI_6-4_, the edge is higher than the center (Figure 1(a), also see Figure S4). This is due to the proximity effect in electron-beam lithography (EBL), where the actual exposure dose at the center of the pattern is higher [44, 45]. This phenomenon is consistent with the PL intensity of various squares changing with the e-beam dose. Meanwhile, the PL emission peak demonstrates a successive blue shift from 762 nm to 752 nm and gradually stabilized at 750 nm (Figure 1(b)). Specifically, as shown in Figures 1(c) and 1(d), the PL emission peak blue-shifts sharply from 762 nm (MAPI_0_) to 753.7 nm (MAPI_4-1_), then moves slowly to 752 nm (MAPI_9-1_), and eventually stabilizes at 750 nm. The enhanced PL intensity together with the blue-shift of PL emission peak implies a suppressed nonradiative recombination due to the reduced surface defects of the MAPI films [46–50].

**Figure 2:**
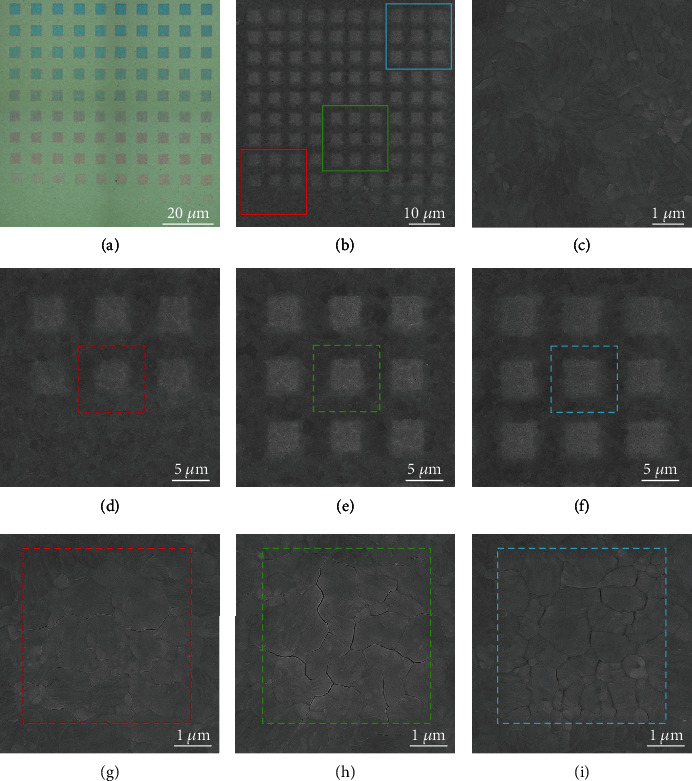
Morphological changes of MAPI films upon e-beam irradiation. (a) Optical image of MAPI_1-1_ to MAPI_10-10_. MAPI shows different colors with various e-beam dose treatment. (b) SEM image of MAPI_1-1_ to MAPI_10-10_. 3 × 3 arrays centered at MAPI_2-2_, MAPI_6-4_, and MAPI_9-9_ are marked with red, green, and blue box. (c) SEM image of MAPI_0_ with grains densely packed. (d–f) Corresponding magnified SEM images of arrays shown in (b). (g–i) Corresponding magnified SEM images of MAPI_2-2,_ MAPI_6-4_, and MAPI_9-9._

To fully explore the interaction between e-beam and MAPI films, we carefully analyzed the structural and compositional changes of the MAPI films after e-beam exposure.  Figure 2(a) shows the optical micrography of MAPI films irradiated by varying e-beam dose. As the optical reflection is heavily dependent on the film thickness, various colors will be controllably produced depending on the exposure dose. The relationship between the thinning of the MAPI film thickness and the e-beam dose will be explained in detail later. Direct writing of structural colors together with the high spatial resolution afforded by EBL has many potential applications in optical anticounterfeiting, fade-resistant color printing, and colorimetric sensing [51–54]. To investigate the dependence of surface morphology of MAPI films on the dose, top-view and cross-sectional SEM images were conducted. As illustrated in Figure 2(b), the drawn patterns of the 10 by 10 array can be clearly seen by the contrast of the image, which display brighter colors. MAPI_0_ without e-beam treatment is smooth with crystal grains densely packed (Figure 2(c)). We have selected three representative regions, 3 × 3 arrays centered at MAPI_2-2_ (initial stage, Figure 2(d)), MAPI_6-4_ (PL_max_, Figure 2(e)), and MAPI_9-9_ (final stage, Figure 2(f)), and marked with red, green, and blue box in Figure 2(b) for detail analysis. As shown in Figure 2(g), cracks begin to appear along the grain boundaries and then extended across the full exposure area (Figures 2(h) and 2(i)), leaving numerous smaller grains. The width of the cracks can be extended to *ca.* 20 nm, which will impede charge carrier transport and thus deteriorate device performance. Furthermore, atomic force microscope (AFM) was conducted to measure the height profile and roughness of the e-beam treated films. As shown in Figure S4, AFM images reveal the thinning of the MAPI films upon e-beam exposure. The thickness of the as-prepared MAPI films is about 330 nm (Figure S5). The thickness thinning of MAPI_2-2_, MAPI_6-4_, and MAPI_9-9_ is about 25 nm, 30 nm, and 45 nm (Figure S4b-g), respectively. The gradual thinning of MAPI films leads to the blue shift of the color displayed in optical microscope as mentioned above. Moreover, the roughness of these three regions is 8.3 nm, 9.4 nm, and 9.5 nm, which is almost the same as that of MAPI_0_ (8.2 nm). These changes in morphology provide evidence for the e-beam-driven directional “etching” of the MAPI films, which may have potential applications in fabrication of micro/nanostructured perovskite films.

**Figure 3:**
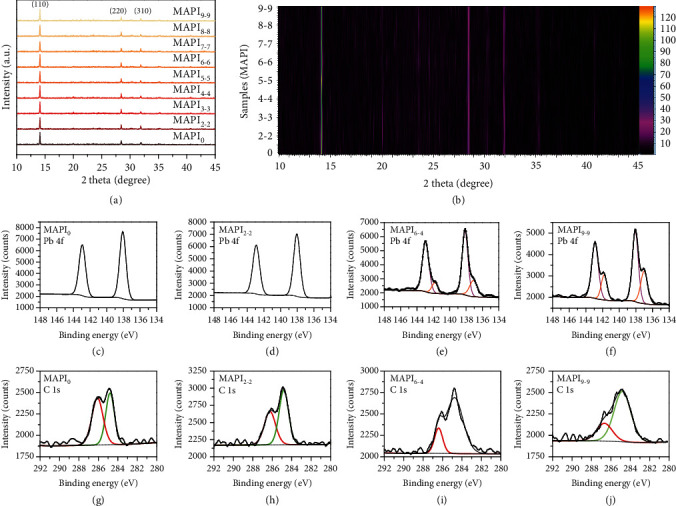
XRD and XPS Characterizations. (a) XRD patterns of MAPI_0_, MAPI_2-2_, MAPI_3-3_, MAPI_4-4_, MAPI_5-5_, MAPI_6-6_, MAPI_7-7_, MAPI_8-8_, and MAPI_9-9_. (b) Corresponding contour map of XRD patterns. (c–f) Pb 4f spectra of MAPI_0_, MAPI_2-2_, MAPI_6-4_, and MAPI_9-9_. (g–j) C 1s spectra of MAPI_0_, MAPI_2-2_, MAPI_6-4_, and MAPI_9-9_.

A thorough analysis of the compositional change of MAPI films upon e-beam exposure was carried out to further explore the underlying mechanism of the enhanced PL emission. The energy-dispersive X-ray spectroscopy (EDS) was firstly used to record the element content of MAPI films (Figure S6). The I/Pb ratio of MAPI_0_ is 60.7/18.8, which agrees well with the stoichiometry of PbI_3_. Upon e-beam irradiation, I and Pb intensity do not change appreciably, with I/Pb ratio of 60.4/19.2, 60.5/19.4, 59.9/19.4, 61.4/18.2, and 60.0/19.5 for MAPI_1-1_, MAPI_3-3_, MAPI_5-5_, MAPI_7-7_, and MAPI_9-9_, respectively, suggesting that the I/Pb ratio is almost independent of the e-beam dose. We then carried out high-resolution thin-film X-ray diffractometer (HRXRD) for MAPI films to study the effect of e-beam irradiation on the crystal structure.  Figure 3(a) shows three prominent peaks at 14.1°, 28.5°, and 31.9°, corresponding to the (110), (220), and (310) planes, which demonstrate robust tetragonal phase of MAPI films. Intensities of these three peaks are also well preserved as shown in Figure 3(b). It is noteworthy that MAPbI_3_ would suffer from thermal degradation and convert into PbI_2_ upon temperature increase. The absence of PbI_2_-related peaks in the XRD spectra (at scattering angle of 12.7°) indicates that the thermal effect is negligible in our experiments. Combining EDS and XRD measurement, we conclude that the bulk composition and crystal structure of MAPI films have not changed upon e-beam exposure in our case.

We then carried out X-ray photoelectron spectroscopy (XPS) to explore the composition on the surface of MAPI films. Figures 3(c)–3(j) show the evolution of XPS Pb 4f and C 1s core-level spectra as a function of e-beam dose. Two-sharp peaks pointing to Pb 4f_7/2_ at 138.0 eV and Pb 4f_5/2_ at 143.0 eV in MAPI_0_ can be assigned to the Pb^2+^ (Figure 3(c)). The Pb 4f peaks are well preserved in MAPI_2-2_ (Figure 3(d)). However, the continuous e-beam exposure caused the reduction of Pb^2+^ to metallic Pb in MAPI_6-4_ with the appearance of Pb^0^ peaks (Figure 3(e)). The decreased and enhanced intensities of Pb^2+^ and Pb^0^ peaks as the dose increased to MAPI_9-9_ (Figure 3(f)) suggest the persistent reduction of Pb^2+^ to metallic Pb through radiolysis. Figures 3(g)–3(j) show the C 1s core-level spectra of MAPI_0_, MAPI_2-2_, MAPI_6-4_, and MAPI_9-9_, respectively. The higher binding energy at 286.3 eV represents the C-N bond attributed to CH_3_NH_3_ cation, and the other lower binding energy at 284.8 eV results from the absorbed ex situ hydrocarbons (e.g., C-C, C-H) [55]. It is obvious that the peak intensity of C-N bond gradually diminishes with increasing e-beam dose, suggesting the persistent decomposition of CH_3_NH_3_ cation.

**Figure 4:**
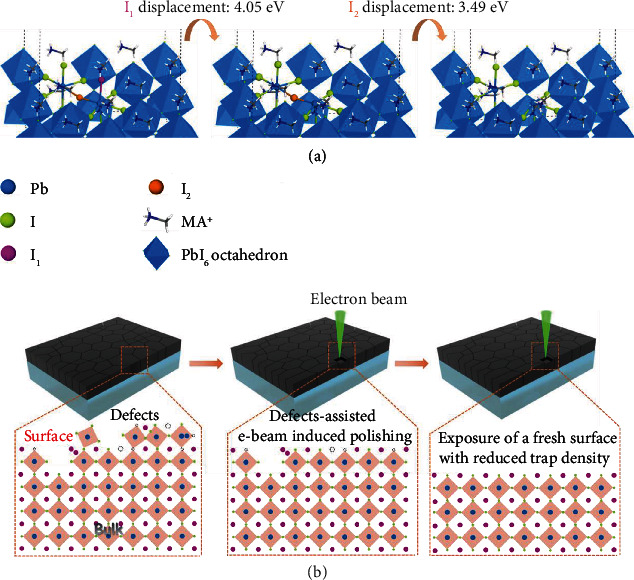
The mechanism for the regulation of surface defects of MAPI films via e-beam irradiation. (a) Calculated activation energies for I ion displacement (*E*_a_) on perfect slab and *V*_I_ slab, *E*_a_ for I_1_ and I_2_ are 4.05 eV and 3.49 eV. (b) A schematic illustration for the regulation of surface defects, e-beam induced thinning of MAPI films through knock-on assisted by surface defects.

Till now, the experimental results gradually reveal the interaction between the e-beam and MAPI films. It is known that knock-on damage and radiolysis usually occur during electron-matter interaction [42, 43]. When the electron energy is beyond a certain threshold energy for the displacement (*E*_d_) of a particular atom, knock-on damage and surface sputtering will happen. Radiolysis (or ionization damage) involves inelastic electron-electron or electron-phonon scattering that drives atomic displacement through energy-momentum transfer assisted by either thermal vibrations or Coulomb interactions. In previous studies, e-beam induced radiolysis has been considered as the major mechanism in perovskite materials since the incident electron energy is usually below the *E*_d_ [41]. In reality, such a threshold can be significantly lowered because of the finite sample size (weak bonds of surface and edge atoms) and the existence of defects [56]. Therefore, the knock-on effect cannot be neglected. In this study, considering the soft ionic nature of MAPI films, various defects such as point defects (i.e., vacancies, interstitials, and antisites) and two-dimensional defects (grain boundaries) are generated unavoidably due to the fast annealing crystallization process and solution-processed stoichiometric issues [57]. Moreover, the trap states are 1-2 orders of magnitude greater than that of the film interior [58]. We calculated the activation energies for the displacement of halide ions on MAPI surfaces with and without halide vacancies (*V*_I_). The activation energy for I ion displacement in defect-free MAPI surface was 4.05 eV; whereas a lower activation energy of 3.49 eV was found on *V*_I_ surface (Figure 4(a)). This indicates that *V*_I_ could reduce the threshold for the following atomic displacement. We speculate that defect-assisted knock-on might first take place during e-beam exposure, just like the ripple effect, leading to the thinning of MAPI film and the emergence of underlying fresh MAPI (Figure 4(b)). The fresh surface with much less defect density than initial MAPI surface contributed to the enhanced PL emission and blue-shifted emission peak. Meanwhile, the radiolysis induced decomposition of C-N bond and reduction of Pb^2+^ to metallic Pb was accompanied with knock-on, which deteriorated MAPI crystal structures and thus weakened PL emission. It is notable that both knock-on and radiolysis are thought to be dependent only on total dose received and independent on dose rate. Therefore, the final PL emission is the result of the balance of these two effects. With increasing the e-beam dose from MAPI_0_ to MAPI_6-4_, knock-on is stronger than radiolysis, leading to the persistent increasing in PL emission. However, the radiolysis plays a dominant role with continuously increasing dose; as a result, the PL intensity begins to decrease and finally lowers than that of MAPI_0_. The efficiency of radiolytic damage is strongly temperature dependent, and the damage rate can be significantly reduced when cooling the specimen. In the future, we will further explore the PL emission of MAPI films upon e-beam irradiation at low temperature.

**Figure 5:**
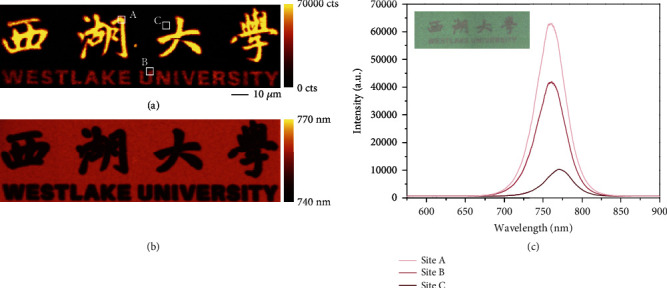
E-beam writing micropatterns on MAPI films. (a) PL intensity mapping of “Westlake University”. The dose for sites A, B, and C is 8800, 4050, and 0 *μ*C/cm^2^. (b) PL emission peak mapping of “Westlake University.” (c) Corresponding PL spectra of sites A, B, and C, respectively. Inset is an optical image of “Westlake University”.

Taking advantage of e-beam regulating surface defects, we demonstrate a high-resolution patterned MAPI film with controllable PL emission. As illustrated in Figures 5(a) and 5(b), the patterns of “WESTLAKE UNIVERSITY” in Chinese and English characters have different PL intensities and peaks. The exposure doses for sites A, B, and C are 8800 *μ*C/cm^2^ (equivalent to MAPI_6-4_), 4050 *μ*C/cm^2^ (equivalent to MAPI_7-2_), and 0 (equivalent to MAPI_0_), respectively, and their PL emission spectra are shown in Figure 5(c) with an optical image inset. This patterning method has potential applications in fabricating perovskite-based micro-LED display, which is widely believed to be incompatible with typical top-down lithography [59].

**Figure 6:**
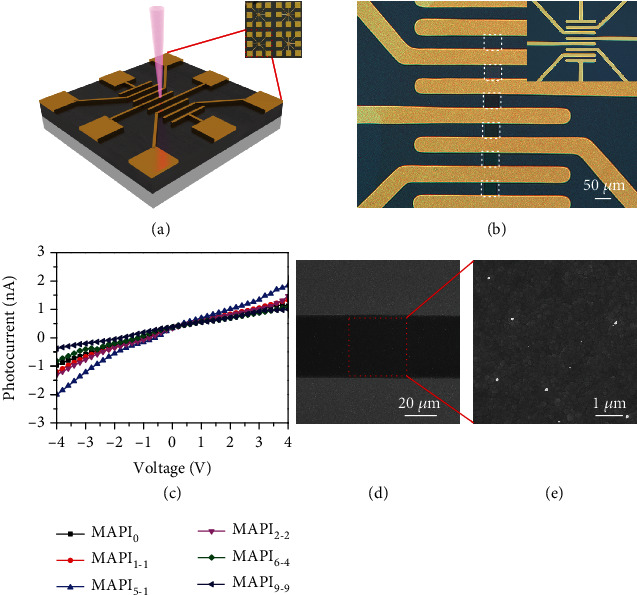
Structure and characteristics of fabricated MAPI photodetectors. (a) Schematic illustration of the photodetector. (b) Optical image of the MAPI photodetector upon e-beam irradiation (marked with dashed line box). Inset is the original photodetector. (c) I-V characteristics of MAPI_0_, MAPI_1-1_, MAPI_5-1_, MAPI_2-2_, MAPI_6-4_, and MAPI_9-9_ with 0.5 mW/cm^2^ halogen light illumination. (d, e) SEM image and corresponding magnified image of MAPI_5-1_.

The e-beam induced defect regulation in MAPI film is also applied to improve the performance of optoelectronic devices. Here, we fabricated a metal-semiconductor-metal (MSM) MAPI photodetector (PD) as an example. 60 nm thick gold electrodes with a 40 *μ*m electrode gap were deposited on MAPI films by shadow mask-assisted e-beam evaporation. The schematic illustration of PD is shown in Figure 6(a). Then, the MAPI film was partially treated through e-beam irradiation with various doses. An optical image in Figure 6(b) clearly shows exposed areas between electrode gaps. Figure 6(c) shows I-V curves of our fabricated PD under halogen light illumination with irradiance of 0.5 mW/cm^2^. The spot radius is *ca.* 10 *μ*m and it is confined within the e-beam exposure region. For the photodetector without e-beam irradiation, we obtain a photocurrent of 1.0 nA when applying a voltage of 4 V. As expected, the photocurrent can be raised after e-beam exposure, and the maximum is about 2.0 nA using the dose as MAPI_5-1_. From the SEM image in Figures 6(d) and 6(e), we see that grains in the MAPI film are still densely packed. The improved photocurrent is attributed to the less trap states at the MAPI surface and the following decrease is due to the broadened grain boundaries which make the charge carries encounter more scattering. In terms of their being unstable in common humid air, a passivation using a hydrophobic polymer layer or thin glass encapsulation is necessary for practical applications.

## 3. Conclusions

In summary, we have experimentally investigated structural and compositional changes of MAPI films upon e-beam irradiation with various doses. Based on theories of electron-matter interaction and careful characterizations of surface morphology, crystalline, element composition, and valence, we believe that the counterintuitive PL enhancement after e-beam exposure is attributed to the emergence of a fresh surface with less defects. The e-beam regulating surface defects promotes the fabrication of a patterned MAPI film with controllable PL and a photodetector with enhanced photocurrent. This work provides an alternative way to precisely regulate and improve the performance of OIHP devices with typical electron beam lithography.

## 4. Experimental Methods

### 4.1. Materials

Methylammonium iodide (MAI, 99.5%) and lead iodide (PbI_2_, 99.999%) were purchased from Shanghai MaterWin New Materials Co., Ltd. N, N-dimethylformamide (DMF, 99.7+%), dimethyl sulfoxide (DMSO), and Chlorobenzene were purchased from Alfa Aesar. All materials and reagents were used as received without further purification.

### 4.2. Synthesis MAPI Film

The MAPI film was prepared by a typical antisolvent spin-coating method. Specifically, the MAPI perovskite ink (1.25 M) was prepared by dissolving MAI and PbI_2_ in a mixture solvent of DMF and DMSO (4 : 1, *v*/*v*). 40 *μ*L of MAPI precursor were dropped on the ITO glass, followed by spinning at 6000 rpm for 30 s. At the 6^th^ second of spinning, 200 *μ*L of chlorobenzene was quickly dropped to assist in forming a dense perovskite film and thermal annealing at a temperature of 100°C on the hotplate was to crystallize the MAPI film.

### 4.3. Photodetector Fabrication

MAPI films were prepared on a 100 nm SiO_2_-covered Si wafer as described previously. A 60 nm thick gold film was deposited on perovskite surface by e-beam evaporator using a shadow mask. The gap between the electrode pairs is 40 *μ*m. Then, the gap was performed with e-beam irradiation (acceleration voltage: 10 kV; current beam: 200 pA) with various doses.

### 4.4. DFT Calculations

The DFT calculations were performed by the CASTEP package [60] with the ultrasoft pseudopotentials [61] and GGA-PW91 functional [62, 63]. A 600 eV plane wave basis sets the cutoff, and the cutoff energy was chosen in our calculations. The 3 × 3 × 1 Monkhorst-Pack grids [64] of *k*-points were used for the (001) surface of tetragonal MAPbI_3_ phase. The convergence thresholds between optimization cycles for energy change and maximum force were set as 5.0 × 10^−6^ eV/atom and 0.03 eV/Å, respectively.

A stoichiometric slab of 17.68 Å × 17.68 Å × 24.84 Å with a vacuum thickness of 12 Å was constructed to model (001) surface of tetragonal MAPbI_3_ phase. The MAI-terminal was selected. In all calculations, the atoms in the bottom layers were fixed, but the atoms in the two topmost layers were allowed to relax. The formation energy of iodine defects was defined as

(1) $$\begin{array}{c} E_{\text{f}}=\left[E_{\left(\text{iodine}\right)}+E_{\left(\text{defect slab}\right)}\right]-E_{\left(\text{perfect slab}\right)}, \end{array}$$

where the first term is the total energy of isolated iodine atom, molecule, the second term is the total energy of defect slab containing iodine defect, and the third term is the total energy of the perfect slab without defect. According to above definitions, a larger *E*_f_ value represents that the iodine defect is more difficult to emerge.

### 4.5. Characterization

UV–vis–NIR absorption of MAPI films was recorded on the UV3600Plus spectrophotometer (Shimadzu, Japan). Steady-state photoluminescence (PL) spectra and images were measured on alpha 300R (WITec GMBH, Germany) confocal Raman system. A diode-pumped solid-state laser (532 nm, cobalt Laser) was focused on samples with a diffraction-limited beam size of 350 nm by a 100x objective (NA = 0.90). The collected PL signal was dispersed by UHTS 600 mm spectrometer and detected using an electron-magnified charge-coupled-device (EMCCD) thermoelectrically cooled to -60°C. Ultrafast PL imaging was running with 100 nm step size and 15 ms integration time. The time-resolved photoluminescence spectra were recorded using standard time-correlated single-photon counting (TCSPC) Lifetime fluorescence spectrometer (FLS1000, Edinburgh, UK). The exciting light was a picosecond pulsed laser source at 405 nm (EPL-405, Pulse Width 46.7 ps). The XRD patterns were recorded on a desktop diffractometer with a Cu K*α* source at the range of 10°–60° (D8 Advance; Bruker, Germany). The microdomain XRD analysis was performed on Bruker D8 Discover High-Resolution X-ray diffractometer, which is equipped with point-focused rotational Cu anode and DUO detectors of Scintillation counter and LynxEye detector. Each individual domain was precisely localized by laser-assisted camera and well aligned by procedure of z scan for sample height determination and rocking curve for sample surface alignment. Then, measurements were conducted by 2Theta-Omega scan or Grazing Incident Diffraction (GID). XPS was performed on ESCLAB Xi+ (Thermo Fisher), using a monochromatized X-ray source (Al K*α*, 1486.6 eV). The diameter of the incident X-ray spot was 200 *μ*m. Analysis area of 100 *μ*m was obtained by reducing apertures. Location of microdomain was confirmed by parallel imaging. Energy calibration was performed by fixing the C-C component of C 1s spectrum at 284.8 eV. SEM images and EDS analysis of the samples were obtained on a field emission scanning electron microscope (Crossbeam550L, Zeiss) with an accelerating voltage of 10 kV.

## Data Availability

All data are available in the manuscript, supplementary materials, or from the author.

## Supplementary Materials

Supplementary Materials Figure S1: (a) XRD pattern, (b) UV-vis absorbance spectrum, (c) steady PL spectrum, and (d) time-resolved PL spectrum of MAPI0 sample. Figure S2: distribution of E-beam dose factor from MAPI1-1 to MAPI10-10. Figure S3: steady PL spectra of MAPI0, MAPI1-1, MAPI2-2, MAPI3-3, MAPI4-4, MAPI5-5, MAPI6-6, MAPI7-7, MAPI8-8, MAPI9-9, and MAPI10-10, respectively. Figure S4: PL intensity extract from (a) MAPI6-2, MAPI7-2, and MAPI8-2 and (b) MAPI6-8, MAPI7-8, and MAPI8-8. Figure S5: AFM image of (a) MAPI0, three representative 3∗3 array centered at (b) MAPI2-2, (c) MAPI6-4, and (d) MAPI9-9. Height profile of (e) MAPI2-2 marked in (b), (f) MAPI6-4 marked in (c), and (g) MAPI9-9 marked in (d). Figure S6: cross-sectional SEM image of MAPI film coated on ITO glass.
