# Supplementary material for: Electron-Beam Irradiation Induced Regulation of Surface Defects in Lead Halide Perovskite Thin Films
Source: Research (Wash D C). 2021 Jun 2;2021:9797058. doi: 10.34133/2021/9797058 (PMC8214510; doi:10.34133/2021/9797058)
Supplement: Supplementary Materials — Figure S1: (a) XRD pattern, (b) UV-vis absorbance spectrum, (c) steady PL spectrum, and (d) time-resolved PL spectrum of MAPI0 sample. Figure S2: distribution of E-beam dose factor from MAPI1-1 to MAPI10-10. Figure S3: steady PL spectra of MAPI0, MAPI1-1, MAPI2-2, MAPI3-3, MAPI4-4, MAPI5-5, MAPI6-6, MAPI7-7, MAPI8-8, MAPI9-9, and MAPI10-10, respectively. Figure S4: PL intensity extract from (a) MAPI6-2, MAPI7-2, and MAPI8-2 and (b) MAPI6-8, MAPI7-8, and MAPI8-8. Figure S5: AFM image of (a) MAPI0, three representative 3∗3 array centered at (b) MAPI2-2, (c) MAPI6-4, and (d) MAPI9-9. Height profile of (e) MAPI2-2 marked in (b), (f) MAPI6-4 marked in (c), and (g) MAPI9-9 marked in (d). Figure S6: cross-sectional SEM image of MAPI film coated on ITO glass. [file 9797058.f1.docx]

Supplemental Information

Electron-beam irradiation induced regulation of surface defects in lead halide perovskite thin films

Binbin Jin,^1, 2^ Ding Zhao,^1, 2, *^ Fei Liang,^3^ Lufang Liu,^4^ Dongli Liu,^1, 2^ Pan Wang,^4^ Min Qiu^1, 2, *^

1Key Laboratory of 3D Micro/Nano Fabrication and Characterization of Zhejiang Province, School of Engineering, Westlake University, 18 Shilongshan Road, Hangzhou 310024, Zhejiang Province, China.

2Institute of Advanced Technology, Westlake Institute for Advanced Study, 18 Shilongshan Road, Hangzhou 310024, Zhejiang Province, China.

3State Key Laboratory of Crystal Materials and Institute of Crystal Materials, Shandong University, Jinan 250100, China

4State Key Laboratory of Modern Optical Instrumentation, College of Optical Science and Engineering, Zhejiang University, Hangzhou 310027, China.


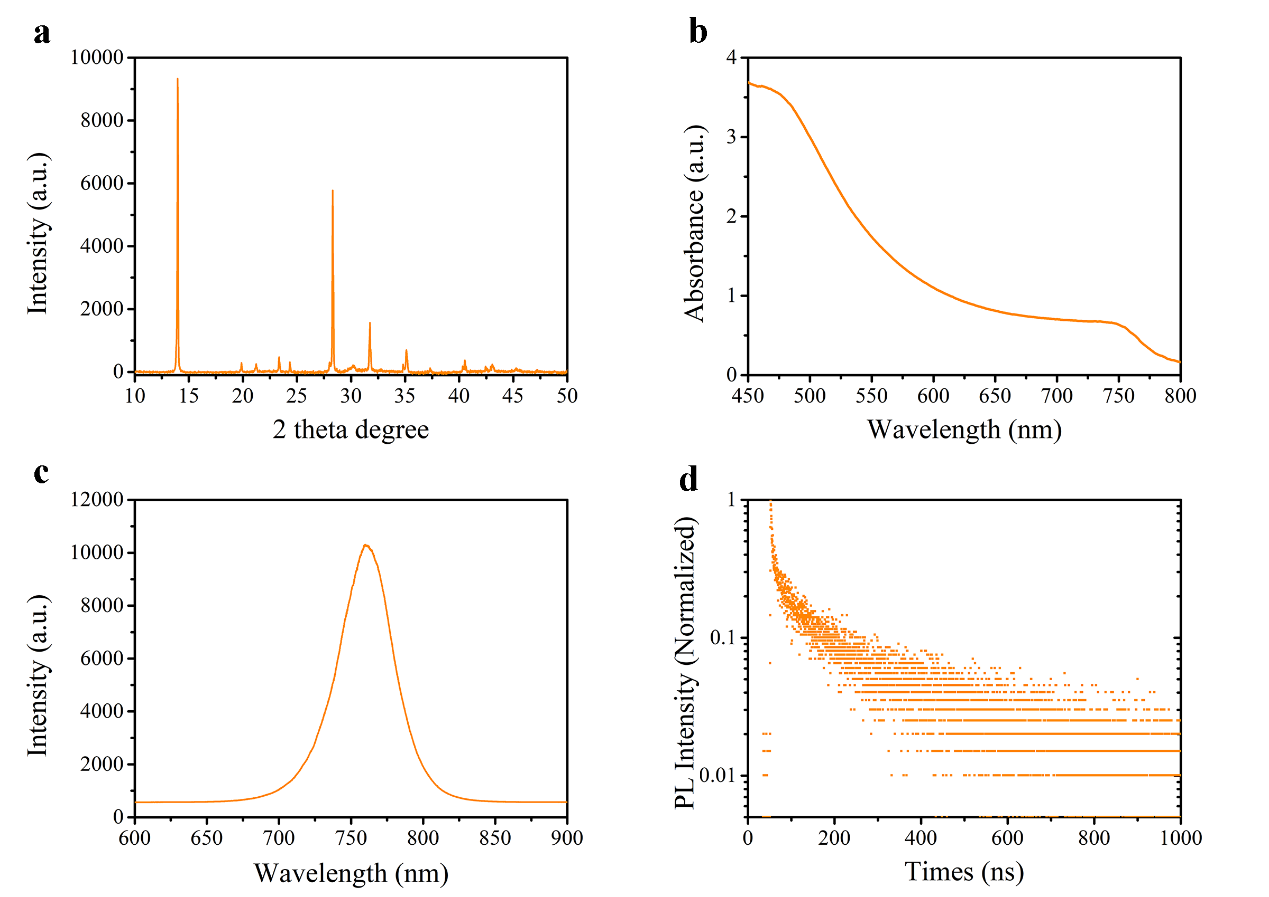


Figure S1. (a) XRD pattern, (b) UV-vis absorbance spectrum, (c) steady PL spectrum, and (d) time-resolved PL spectrum of MAPI_0_ sample.


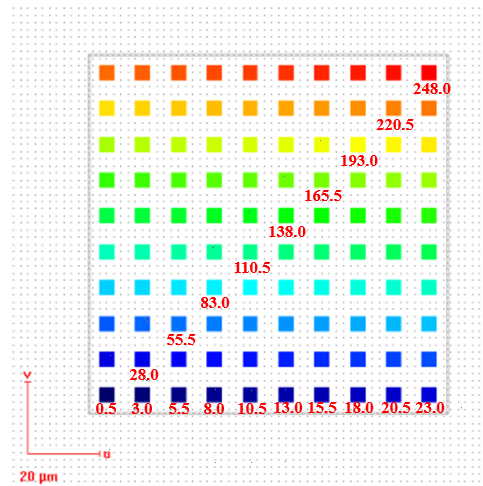


Figure S2. Distribution of E-beam dose factor from MAPI_1-1_ to MAPI_10-10_.





Figure S3. Steady PL spectra of MAPI_0_, MAPI_1-1_, MAPI_2-2_, MAPI_3-3_, MAPI_4-4_, MAPI_5-5_, MAPI_6-6_, MAPI_7-7_, MAPI_8-8_, MAPI_9-9_ and MAPI_10-10_, respectively.
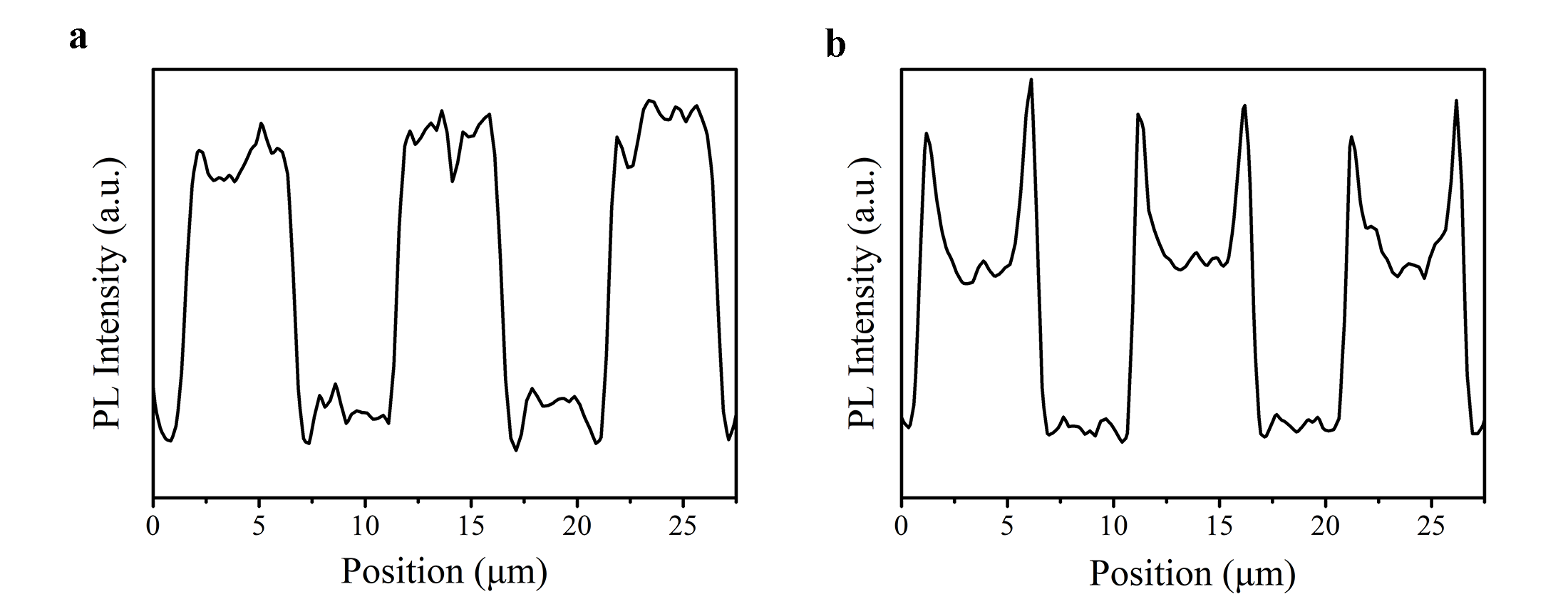


Figure S4. PL intensity extract from (a) MAPI_6-2_, MAPI_7-2_, MAPI_8-2_, and (b) MAPI_6-8_, MAPI_7-8_, MAPI_8-8_.





Figure S5. AFM image of (a) MAPI_0_, three representative 3*3 array centered at (b) MAPI_2-2_, (c) MAPI_6-4_, and (D) MAPI_9-9_. Height profile of (e) MAPI_2-2_ marked in Figure S5b, (f) MAPI_6-4_ marked in Figure S5c, and (g) MAPI_9-9_ marked in Figure S5d.


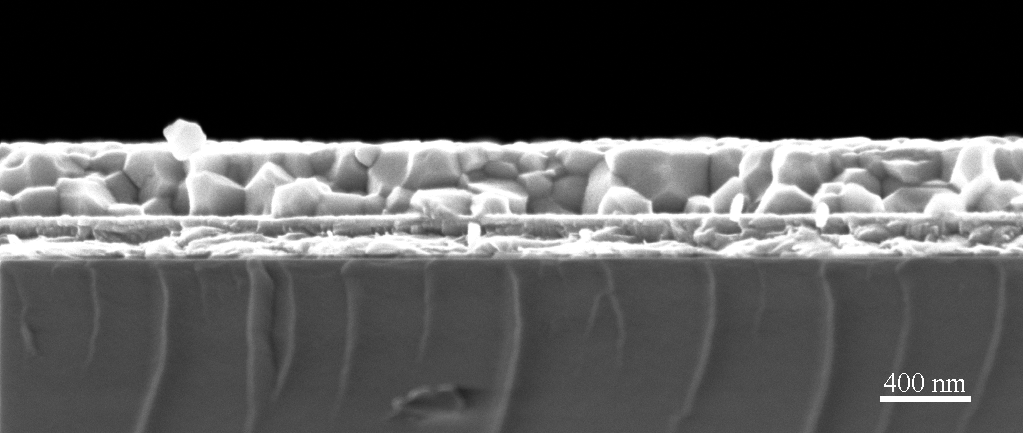


ITO

MAPI

Figure S6. Cross-sectional SEM image of MAPI film coated on ITO glass.


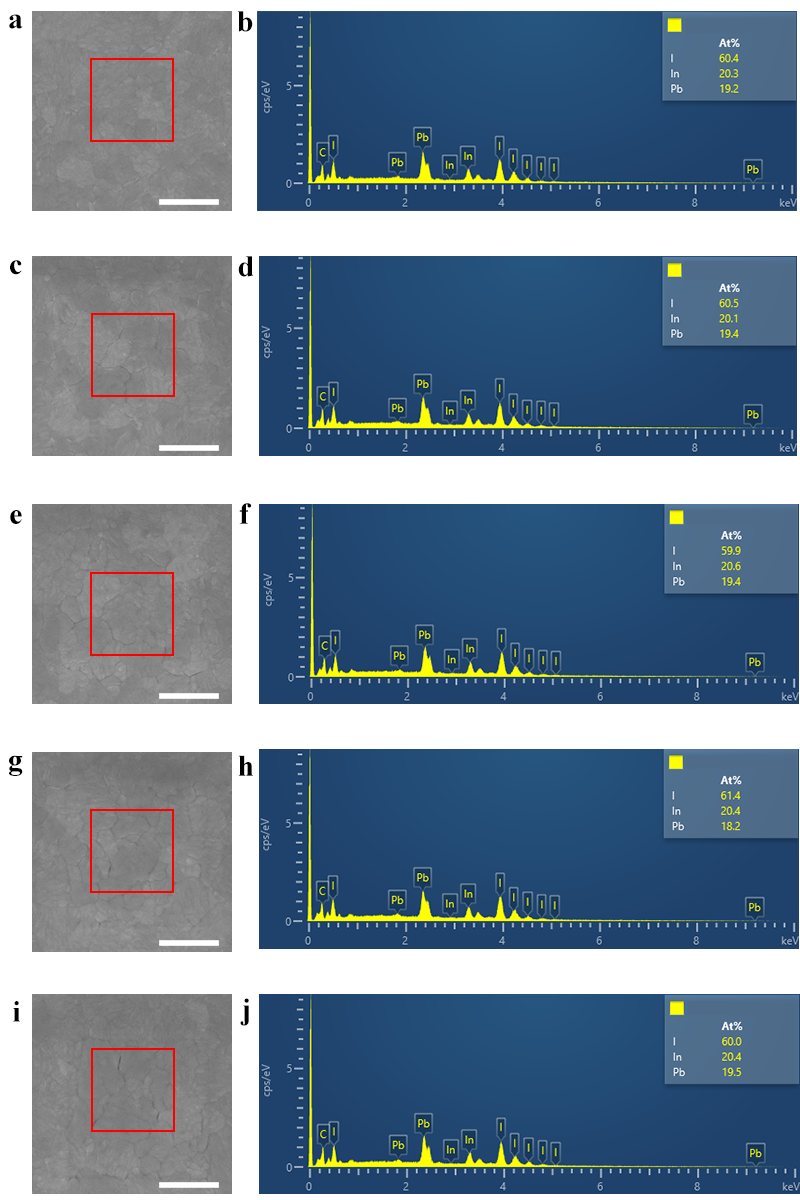


Figure S7. SEM images and EDS spectra of (a, b) MAPI_1-1_, (c, d) MAPI_3-3_, (e, f) MAPI_5-5_, (g, h) MAPI_7-7_ and (i, j) MAPI_9-9_, respectively.
